# Macrophages and glial cells: Innate immune drivers of inflammatory arthritic pain perception from peripheral joints to the central nervous system

**DOI:** 10.3389/fpain.2022.1018800

**Published:** 2022-10-26

**Authors:** Kaue Franco Malange, Juliana M. Navia-Pelaez, Elayne Vieira Dias, Julia Borges Paes Lemes, Soo-Ho Choi, Gilson Goncalves Dos Santos, Tony L. Yaksh, Maripat Corr

**Affiliations:** ^1^ Department of Anesthesiology, University of California, San Diego, CA, United States; ^2^ Department of Medicine, University of California, San Diego, CA, United States; ^3^ Department of Neurology, University of California, San Francisco, CA, United States

**Keywords:** arthritis, pain, macrophages, glial cells, central mechanisms, peripheral mechanisms, treatments

## Abstract

Millions of people suffer from arthritis worldwide, consistently struggling with daily activities due to debilitating pain evoked by this disease. Perhaps the most intensively investigated type of inflammatory arthritis is rheumatoid arthritis (RA), where, despite considerable advances in research and clinical management, gaps regarding the neuroimmune interactions that guide inflammation and chronic pain in this disease remain to be clarified. The pain and inflammation associated with arthritis are not isolated to the joints, and inflammatory mechanisms induced by different immune and glial cells in other tissues may affect the development of chronic pain that results from the disease. This review aims to provide an overview of the state-of-the-art research on the roles that innate immune, and glial cells play in the onset and maintenance of arthritis-associated pain, reviewing nociceptive pathways from the joint through the dorsal root ganglion, spinal circuits, and different structures in the brain. We will focus on the cellular mechanisms related to neuroinflammation and pain, and treatments targeting these mechanisms from the periphery and the CNS. A comprehensive understanding of the role these cells play in peripheral inflammation and initiation of pain and the central pathways in the spinal cord and brain will facilitate identifying new targets and pathways to aide in developing therapeutic strategies to treat joint pain associated with RA.

## Introduction

Arthritis is a leading cause of disability worldwide. Every year, the prevalence of this disease rises, reaching more than 350 million people around the world (https://globalranetwork.org/project/disease-info/). This growing incidence is tied to an increase in life expectancy and aging of the population (1), and a lack of accessibility to effective therapies (2). Although several types of disease-related arthralgia affect people, rheumatoid arthritis (RA) joint pain is a significant cause of disability, affecting daily activities and increasing the global disease burden (3, 4).

RA is a chronic inflammatory disease, and despite advances that have remitted inflammation or delayed the progression of bone and cartilage destruction in patients, chronic pain in RA can persist and is still the primary symptom for which patients seek medical care (5–8). As a multifactorial disease, the debilitating chronic pain phenotype evoked relies on complex neuroimmune interactions running from the peripheral tissue through the dorsal root ganglia (DRG), spinal cord, and brain (9–11). Thus, patient outcome may be improved through a multiple-target approach, treating disease injury and inflammation at different levels to relieve the chronic pain state (12–14).

Given the inflammatory component of RA, innate immune cells such as macrophages and glial cells may have a critical role in the maintenance of disease phenotype, contributing to the development of damage signaling responses involved in tissue degeneration and nociceptive plasticity along the pain pathway (15–21). Reviewing the known pathophysiology of these key cell types involved in the onset and progression of RA and studying how immune and neuroimmune interactions dictate the degenerative phenotype can help identify knowledge gaps that warrant further investigation and potentially identify disease targets.

Thus, the objective of this review is to provide relevant aspects of macrophage and glial cell participation in arthritis physiopathology, giving particular focus on the role of these cells in inflammation and the development of chronic pain. We give special attention to key tissue/locations along the nociceptive pathway in arthritis, focusing on the synovium, dorsal root ganglia, spinal cord, and brain to discuss and highlight the role of these cells in the influence on the disease-pain phenotype.

### Peripheral nerve innervation of the joint

The sensory perception from joints relies on a strictly controlled neural network to coordinate the dynamic interaction of limbs and body movements (22). Joint innervation spans a broad spectrum, with termini reaching the capsule, synovium, meniscus, ligaments, fat pad, subchondral bone, and periosteum (23, 24). Four types of nerve fibers are thought to conduct sensory information on the physiological and/or noxious stimuli of the joints: Aβ, Aδ, C, and sympathetic nerve fibers (23–25).

Corpuscular endings from Ruffini, Golgi, and Pacini types, originating from Aβ fibers, innervate the capsule, menisci, ligaments, and adjacent periosteum; whereas Aδ and C fibers have free nerve endings (non-corpuscular) in capsules, adipose tissue, ligaments, menisci, periosteum, and synovia, while cartilage is devoid of any type of innervation (23, 25–28). In addition to Aβ, Aδ, and C fibers, sympathetic innervation has a significant density on ligaments, tendons, and capsules, as well as blood vessels in the joint compartment (24, 29).

Physical joint mechanics generate shear forces capable of inducing conformational changes in ion channels present in the plasma membrane of nerve endings at the joint site, leading to their opening and, subsequently, membrane depolarization (24, 30–32). After depolarization, action potentials further conduct sensory information to the central nervous system (CNS) (24, 27). The difference between innocuous and painful stimulation of joints relies on the physiological tissue working range, where any pressure (static or dynamic) and movement that exceeds the basal resistance can be perceived as painful (25, 28, 33). Along this line, joint noxious stimulation can be triggered by intense pressure applied to a specific area or harmful movements such as twisting or sharp rotation (24, 34, 35). Thus, Aδ and C fibers that innervate the joint will be activated during high threshold stimulation of joints (typically nociceptive); in contrast, Aβ fibers will be recruited during low-threshold stimuli, usually innocuous (23).

RA can affect hands, feet, wrists, elbows, knees, and ankle joints (36). Additionally, pre-clinical models used to study this disease use rodents (37, 38), and a significant incidence of disability in tibiofemoral and metatarsophalangeal joints has been reported in this species (39). The data from these preclinical studies suggest that the soma of most sensory neurons that innervate lower limbs, especially the tibiofemoral and metatarsophalangeal joints, are present in the DRG from spinal segments L1–L5 (40–42).

These sensory afferents neurons present in the DRG also play an active part in inflammatory processes, regulating immune cell function through the release of neuropeptides such as substance P and *Calcitonin gene-related peptide* (*CGRP*) that lead to vascular permeabilization and activation of effector cells from the immune system (43–46). These peripheral sensory neurons help regulate the local tissue inflammatory response and maintain the integrity of the affected limb by augmenting the receptive field for noxious stimuli (27, 30). However, in RA, this transient receptive field enhancement might acquire a consolidated state, as suggested by preclinical studies, where the ongoing inflammatory process in the affected limb promotes nerve damage and axonal sprouting, leading to permanent neuroplasticity, which characterizes the chronic pain phenotype (46–50).

The switch in the pattern of joint innervation has been described as one of the main pillars that sustains the chronic pain phenotype in RA (48–51). Nerve sprouting from both sensory and sympathetic innervation significantly correlates with pain severity and tissue injury in some arthritis experimental models (49). Different genetic or induced animal models of arthritis have been developed to study inflammatory RA and post-traumatic or spontaneous osteoarthritis (OA) (see Table 1). In several of these rodent models, chronic inflammation results in an increase in nociceptive afferent and postganglionic sympathetic axons that can be seen in the synovium (29, 46). In a murine model of monoarticular inflammation induced with intra/periarticular injections of Complete Freund's Adjuvant (CFA), an increased density of nociceptive fibers (CGRP^+^), and sympathetic fibers (TH^+^ and VMAT2^+^) were visualized 25 days after the last CFA injection (50, 51). Additionally, an increase in TH^+^ and CGRP^+^ fiber density persists in the synovia of mice in the chronic phase of the K/BxN model, which is associated with increased glial proliferation in the spinal cord (49). Furthermore, mice with proteoglycan and CFA-induced arthritis were treated with guanethidine (to deplete postganglionic sympathetic terminals) or capsaicin (to destroy TRPV1^+^ sensory terminals) had reduced pain behavior, edema, and arthritis severity (51, 54). This evidence indicates that sympathetic and capsaicin-sensitive afferents regulate disease outcomes through local neuroimmune interactions in the peripheral joint (51, 54).

**Table 1:** Rodent models described in this review.

Type	Model	Mono/polyarticular	Species	Description	Glial/macrophage phenotype	Referencess
Mono-articular inflammation	Complete Freund's arthritis (CFA)	Mono	Mice/rats	CFA is injected in the knee or ankle and may require repeated injections.	↑ Microglial IBA1 reactivity in brain	(52)
	Adjuvant	Poly	Rats	Injection of CFA induces polyarticular arthritis.	↑ SGCs in the DRG. Astrocyte reactivity in SC. ↑Microglial IBA1 reactivity in SC and brain.	(21, 53)
	Proteo-glycan-induced arthritis	Poly	Mice	Induced with 4 weekly injections of cartilage proteoglycan (PG). The 1st and 4th injections are of PG/CFA, and the 2nd and 3rd injections are of PG/IFA. Works best in BALB/c mice.		(54)
RA-like	Collagen induced arthritis (CIA)	Poly	Mice/rats	Immunization with type II collagen (CII) emulsified in complete Freund's adjuvant (CFA) and a boost at 21 days with CII in IFA. Best in DBA/J mice.	↑ Microglial IBA1 and GFAP reactivity in brain.	(55, 56)
	Collagen antibody induced arthritis (CAIA)	Poly	Mice	Induced by injection of a cocktail of 4 monoclonal anti-CII antibodies. May need an injection of LPS at 3–5 days. Best in BALB/c mice.	↑ SGCs activation in the DRG. ↑ Microglial IBA1 reactivity in SC.	(18, 57)
	K/BxN serum transfer	Poly	Mice	One or two injections of sera from arthritic transgenic K/BxN mice generates an acute arthritis that later resolves. Penetrant in most background strains.	↑ Pro-inflammatory macrophages in the DRG. ↑ Microglial IBA1 reactivity in SC.	(58–60)
	TNF-Tg	Poly	Mice	Spontaneous arthritis in mice expressing a human tumor necrosis factor (hTNF) transgene.	↑ Region-specific microglial reactivity in the brain.	(61)
OA-like	Surgically-induced	Mono	Mice/rats	Anterior cruciate ligament transection (ACLT), partial or total meniscectomy, medial meniscal transection	↑ Proinflammatory macrophages in the DRG.	(62)
	Chemically-induced	Mono	Mice/rats	Injection directly into the knee joint of a toxic or enzymatic agent like sodium monoiodoacetate (MIA), collagenase, or papain.	↑Anti-and pro-inflammatory macrophages in the DRG. ↑Microgliosis and GFAP in SC.	(62, 63)

## Macrophages and glial cells in the peripheral joint and DRG

### Macrophage ingress related to inflammatory phenotype and neural outcomes

RA susceptibility to autoimmune mechanisms is associated with genetic risk factors (36, 64). Diagnostic markers reflecting the autoimmune component in this disease include an increase in rheumatoid factor (RF; anti-IgG antibodies) and the presence of anti-citrullinated protein antibodies (ACPAs), which can be measured in sera years before the onset of symptoms (65). These serum biomarkers suggest that the pathogenesis of this disease begins gradually, probably associated with different environmental factors necessary to trigger inflammatory responses (36, 65, 66). Such environmental factors include smoking, aging, western diet, alcohol intake, bacterial and viral infections (36, 64, 67–69). These injurious stimuli can lead to the amino acid conversion of arginine to citrulline (a process called deamination or citrullination) in a range of proteins like histones and tissue matrix proteins (fibronectin, collagen, and fibrinogen), tagging these proteins with neoepitopes for autoimmune responses (65, 70–72). Fibronectin, collagen, and fibrinogen are abundant in joints, and these proteins, when citrullinated, are targeted by ACPAs, perpetuating neuroimmune interactions that contribute to the pathogenesis of joint pain and destruction (10, 55, 73–77).

During the acute phase of RA, ACPAs and other autoantibodies, together with complement activation, induce an inflammatory response characterized by cytokine production, microvascular insult, and synovial vascular leakage, contributing to the synovial inflammation and progression of the disease (36). Increased vascular permeability and chemokines facilitate macrophage recruitment and ingress along with other inflammatory cells migrating from the periphery into the joint that initiate degenerative events resulting in pain and articular dysfunction (77–79). Macrophages are specialized innate immune cells that detect, phagocytose, and destroy harmful organisms and cell debris (80). In addition, they act as antigen-presenting cells (APC) and initiate inflammatory responses by releasing chemokines and cytokines that recruit and activate other cell types (80, 81). These infiltrating macrophages are quintessential to the innate immune system and are pivotal in the maintenance of the pain phenotype (77, 82).

In RA, macrophages are activated locally by tissue debris or inflammatory cytokines produced by synovial cells (78, 83) and can guide the perpetuation of joint inflammation *via* paracrine mechanisms with multiple other cells, including, but not limited to, fibroblast-like synoviocytes (FLS), T-cells, B-cells, and dendritic cells (77, 79, 84). Macrophages perpetuate the inflammatory cascade by releasing pro-inflammatory cytokines such as tumor necrosis factor (TNF), interleukin (IL)-1β, and IL-6 and by triggering fibroblasts to release several chemokines such as CXCL1, CXCL5, CCL2, CCL5, CCL8, and CCL10 that will in turn act by chemoattracting monocytes, macrophages, and neutrophils (77, 85). In addition to chemokines, FLS facilitate macrophage expansion at the injury site releasing colony-stimulating factors (CSF) such as GM-CSF and M-CSF (78). Altogether, the macrophage-FLS interaction sets up a vicious cycle creating a renewing inflammatory process that perpetuates itself along the disease course (78). In RA, infiltrating macrophages are affected continuously by inflammatory stimuli and participate in the development of chronic synovitis, bone erosion, and cartilage destruction (79, 86, 87). Even in OA, infiltrating macrophages affect disease outcomes in patients and in murine models, in which increased macrophages showing pro-inflammatory “M1-like” markers have been reported (88).

The deleterious proinflammatory response of the infiltrating macrophages is counter-regulated by tissue-resident macrophages (TRMs) that have an immune regulatory phenotype that contribute to maintaining or restoring tissue homeostasis (89). Given the dual roles of macrophages in RA, understanding macrophage subset identity and location may help to understand their pathogenic functions through the course of arthritic disease progression (79, 86). Their location within the synovial microarchitecture is essential, and the expression of specific markers is associated with different functional subtypes (79, 86). Indeed, distinct genetic and molecular signatures of infiltrated macrophages in the synovial tissue highlight their diversity (86).

Separate groups have identified at least five different joint-infiltrating macrophage subtypes in arthritis (86, 90–92). Two clusters that have been found in higher proportion in lymphocyte-rich RA synovium compared to OA, which include a Toll-like receptor (TLR)-activated and IL-1β producing pro-inflammatory macrophage population and an interferon (IFN) producing macrophage population (90, 92). One cluster found in OA synovium seems to be enriched for gene patterns of non-activated macrophages (91) and a phagocytic profile. Two additional clusters were not well characterized by specific markers and not associated with a specific activation state, thus suggesting the presence of homeostatic macrophage phenotypes (86, 90, 92).

TRMs, in contrast to monocyte-derived infiltrating macrophages, support the resolution of inflammation and restoration of a homeostatic state (89). A CX3CR1^+^ resident-like lining macrophage population has been recently described in mice to have a barrier function that secludes the inner joint from the synovial cavity, preventing macrophage infiltration (93). This barrier and the resident lining macrophages are disrupted in the K/BxN and CIA mouse models (93). This subset of TRMs encompasses a similar function to TREM2^+^ macrophages described in humans (87). TRMs also express elevated levels of anti-inflammatory mediators such as IL-1 receptor antagonist (IL-1RA) and osteoprotegerin (OPG) that act as negative regulators for pro-inflammatory cytokines and receptor activator of nuclear factor kappa-B ligand (RANKL), preventing inflammatory responses and bone loss (83). TRMs also induce a repair phenotype in FLS (87) and limit the recruitment and activation of other immune cells (93). Therefore, loss of the balance between the anti-inflammatory and barrier-protective phenotypes of TRM and the proinflammatory responses induced by infiltrated macrophage subtypes may contribute to the inflammatory cytokine milieu that is maintained in RA tissue.

A cytokine-rich milieu like in RA synovial tissue can sensitize nociceptive neurons that express TNF, IL- 1β, and IL-6 receptors. These cytokines sensitize the peripheral terminals in part by modulating voltage-gated sodium channels-tetrodotoxin-resistant (NavTTX-R) and Transient Receptor Potential (TRP) channels, and neuronal excitability. IL-1β stimulates the mitogen-activated protein kinase (MAPK) p38 that further phosphorylates NavTTX-R channels, triggering an increase in sodium currents (94, 95). TNF promotes up-regulation of Nav channel isoforms 1.3 and 1.8 on axons and neuronal soma (96). IL-6 receptor interaction promotes downstream activation of Janus kinase-protein kinase C (JAK/PKC), leading to further phosphorylation of TRPV1 and TRPA1 that enhances the excitability of nociceptive neurons (97). Additionally, reactive oxygen species (ROS) released by tissue-resident cells or inflammatory cells, such as nitric oxide (NO), can potentiate IL-6 actions promoting a feedback loop stimulating TRPV1/TRPA1 *via* phospholipase C (PLC) and PKA (98). All these events, together with the tissue edema from vascular leak, lead to disruption of mechanobiological mechanisms and joint dysfunction, which compromises protective mechanisms for avascular tissues such as cartilage (99, 100).

Extensive and persistent inflammation results in tissue remodeling and a thickened growth of synovial tissue as a pannus (101, 102). The pannus is comprised of proliferative FLS and is interspersed with clusters of immune and inflammatory cells. The rheumatoid pannus has significantly elevated levels of matrix metalloproteinases (MMP) and other deleterious molecules released by immune/inflammatory joint cells, which contribute to the degradation of cartilage (101, 102). In addition, the pannus itself can invade the adjacent subchondral bone (78). Once the subchondral bone is exposed, sensorial nerve fibers (SNFs) that innervate the bone are susceptible to chemical (cytokines, prostanoids) and mechanical injury (28). Exposure of these fibers may lead to the expression of nerve injury markers in the DRG, such as ATF-3 (58). Moreover, both mechanical and chemical injury of SNFs can also lead to mitochondrial injury that is thought to maintain nociceptor hyperexcitability by ROS release, and modulating PKC (103, 104).

### Dorsal root ganglia—mechanisms: macrophage ingress, phenotype, associated cytokines

The DRG functional complexity is exemplified by the excitability of the afferent input circuitry of the DRG system (105, 106). In the DRG, the soma of neurons, macrophages, satellite glial cells, and axons of passage are packed into a small and dense environment. This tissue is supplied by a fenestrated vasculature that lies outside the blood-brain barrier (BBB) and is permeable to large molecular weight molecules and antibodies (106), which may contribute to the increased susceptibility of DRG neurons to neurotoxic agents (107). On the other hand, this vascularization and permeability represent an advantage to target cells in the DRG (108). Aside from neurons, recent work has shown that macrophages and satellite glial cells in the DRG play key roles in pain initiation and the maintenance of pain states (62, 109–111).

The critical contribution of macrophages in the DRG, but not those at the nerve injury site, to the initiation and maintenance of the mechanical hypersensitivity which develops after nerve injury, chemotherapy models, and joint pathology has been well described by many investigators (111, 112). As for arthritis, a recent study reported an increase of macrophages in the DRG in the K/BxN serum transfer model of arthritis (59). These cells were pro-inflammatory macrophages showing “M1-like” markers (CD206^−^/CD11c^+^) accompanied by a significant reduction of specialized pro-resolving lipid mediator Maresin-1 (MaR1) (59). Another study using the mono-iodoacetate (MIA)-induced model of OA showed that infiltration of macrophages with a pro-inflammatory phenotype in the DRG is associated with the release of inflammatory cytokines, IL-1β and TNF, contributing to chronic pain behavior (62). In addition, data from a surgically-induced OA model also demonstrate an increase in macrophage ingress to the DRG (62). As in the synovium, TNF, IL-1β, and IL-6 can also be produced locally in DRG by neurons or associated cells, such as macrophages and satellite glial cells (62, 113–116). TNF has been shown to have varied effects on neuronal excitability that do not require alterations in gene transcription but are the results of its action upon ion channels such as voltage-sodium-gated and TRPV1 channels (96, 117). Moreover, TNF has been associated with monocyte chemoattractant protein 1 (MCP1) production, which can induce a spontaneously active profile at resting potential in the DRG neurons (118).

Anti-inflammatory and homeostatic macrophages can repair damaged tissue and release anti-inflammatory cytokines (119, 120). During the resolution of inflammation, macrophages predominantly adopt an anti-inflammatory phenotype previously designated as “M2” (119). In the DRG, these anti-inflammatory tissue-like repair macrophages play a protective role both in neuropathic and inflammatory pain (62, 111, 116, 121). Indeed, the adoptive transfer of M2 polarized macrophages can reduce neuropathic pain (121). A lack of tissue-like repair macrophages was found in the inflammatory phase of the MIA model of OA (62). Recently, a mechanistic study of a homeostatic, anti-inflammatory macrophage population in the DRG expressing CD206 (an “M2-like” marker), modulation has shown that this macrophage population is responsible for the transfer of mitochondria to DRG neurons preventing neuronal damage and that this is critical for producing analgesia *via* a sustained endogenous opioid release (116). Together, these studies show that the maintenance of pain is associated with an imbalance of diverse DRG macrophages and cytokine production (62, 110, 111, 122, 123).

#### Satellite glial cells

The satellite glial cells (SGCs) in the DRG constitute a sheath of elongated cells enwrapping each body of sensory neurons (124). Their nutritional support role in the DRG microenvironment is already well described (124). However, in the past few years, SGCs emerged as key cells in the development of hyperalgesia in diverse painful states, including in arthritic pain (57, 125). In a rat model of arthritis induced by intra-articular injection of CFA, an increased number of SGCs in the DRG was reported, and the inhibition of these cells was able to attenuate the CFA-evoked mechanical hyperalgesia (21). Additionally, elevated levels of glial fibrillary acidic protein (GFAP) expression, a marker of SGCs reactivity, were detected in L4-L5 DRGs of rats with monoarthritis induced by collagenase injection (126, 127). This GFAP overexpression was predominantly found around injured neurons (ATF3^+^ cells) (126, 127).

Due to the tight proximity between neurons and SGCs in the DRG, different mediators are exchanged between them, leading to neuronal sensitization and nociceptive signal modulation (125, 128). Previous evidence demonstrated that SGCs release neurotransmitters in the DRG such as ATP, glutamate, and pro-inflammatory cytokines (124, 129). These mediators have been associated with neuronal hyperexcitability in neuropathic and inflammatory rodent pain models (113, 128, 130, 131). Recently, a study demonstrated pronociceptive neurochemical changes in the DRG in the collagen antibody-induced arthritis (CAIA) model, which was associated with SGCs activity (57). In the late phase of CAIA, there was an up-regulation of lysophosphatidic acid (LPA) in the DRG, which promoted the activation of the SGC-expressed LPA1 receptor. In response to this, SGCs produced elevated levels of cytokines and nerve growth factor (NGF) leading to nociceptor excitability and prolonged sensitization (57).

**Figure 1:**
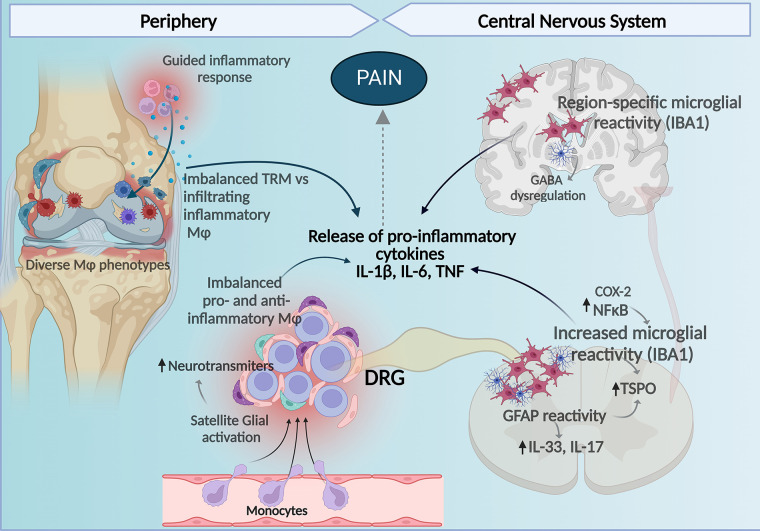
Central and peripheral mechanisms involving macrophages and glial cells contribute to the development of pain in RA. Imbalance between homeostatic TRM and infiltrating macrophages with pro-inflammatory profiles to the synovial tissue contribute to the inflammatory milieu perpetuating inflammation, tissue damage, and bone remodeling affecting nociceptors in the joint. In the DRG, increased proinflammatory macrophages and reduced anti-inflammatory homeostatic/resident-like macrophages contribute to release of pro-inflammatory cytokines and together with satellite glia-induced release of neuromodulation molecules there is an increase in DRG neuronal activity. At the spinal cord increased microglial and astrocyte reactivity also enhance production of pro-inflammatory cytokines and in the brain increased glial reactivity in a region-specific manner regulating cytokine release and GABA neurotransmitter tone affects pain perception in arthritis. Figure created with Biorender.com. Mφ, macrophages; TMR, tissue-resident macrophages.

Although multiple investigative groups have demonstrated the involvement of macrophages and satellite glial cells in several arthritic pain models (Figure 1), the mechanisms correlating the DRGs cell populations and the inflammation in the development and maintenance of pain in arthritis remain ill-defined. Patients reported pain levels often poorly correlate with signs and measures of inflammation (5, 6, 8), suggesting that mechanisms other than peripheral inflammation contribute to chronic pain in RA.

## Spinal glial mechanisms in arthritis

Nociceptive signals generated in the peripheral terminals are introduced into the CNS *via* the presynaptic terminal into the spinal dorsal horn where it synapses with second-order neurons (132). These synapses can be tuned by inflammatory cytokines that are produced by glial cells, leading to sensitization (133). Thus, spinal glial released molecules such as inflammatory cytokines are now considered important mediators of persistent pain states (105, 134). While the pathological spinal mechanisms are still being investigated, enough evidence suggests that neuroinflammation induced by glial activation is a crucial factor in the development of RA-associated pain (135, 136) (Figure 1).

Glia cells in the CNS include non-neuronal cell types: (1) microglia, (2) astrocytes, and (3) oligodendrocytes (137). Microglia are tissue-resident macrophages of the CNS constantly scavenging and pruning plaques, dead cells, synapses, and pathogens (138). However, chronic activation of microglia can be detrimental due to a robust secretion of pro-nociceptive mediators/neuromodulators, including proinflammatory cytokines, chemokines, reactive oxygen species (ROS), and proteases (139). Indeed, accumulating evidence suggests a pivotal role of microglial activation in various pain conditions, including peripheral nerve injury, spinal cord injury, inflammation, chemotherapy, and arthritis (58, 140–145). Nevertheless, new evidence also points to a beneficial role of spinal microglia in pain resolution during neuropathic pain (146).

### Microglia

Different experimental models of arthritis have characterized the spinal cords of the experimental animals with an increase in Ionized calcium binding adaptor molecule 1 (IBA1) as a marker of microgliosis (a response to pathogenic insults with an increased number of microglial cells at the lesion site) (147). For instance, the superficial dorsal horn of mice with K/BxN serum transfer arthritis displayed increased staining of microglial IBA1 at early and late time points in the model (58). Microgliosis has also been described early in the model and at 5 and 10 weeks in an ankle joint with MIA-induced OA in rats (63, 148). In both cases, intrathecal inhibition of microglia with minocycline inhibited mechanical allodynia (63, 148). In addition, in the murine CAIA model, hypersensitivity was correlated with the presence of reactive spinal microgliosis (18). In rats subjected to the collagen-induced arthritis (CIA) model increased levels of IL-1β in cerebral spinal fluid were found. Like the OA model, in CIA rats, prolonged intrathecal administration of microglial inhibitors attenuated the development of mechanical allodynia, decreased microgliosis, and reduced IL-1β levels (55).

Microglia can release IL-1β triggered by inflammasome or TLR signaling after exposure to their endogenous ligands (149). Extracellular high mobility group box-1 protein (HMGB1) is an endogenous TLR4 ligand found in spinal cords (150). Neutralizing HMGB1 with an anti-HMGB1 monoclonal antibody or recombinant HMGB1 A-box peptide, reversed CAIA-induced mechanical hypersensitivity and associated microgliosis in a TLR4 dependent manner (150). This occurred during ongoing joint inflammation as well as during the post-inflammatory phase, indicating that spinal HMGB1 and TLR4 have important functions in pain states that continue even after joint inflammation resolves (150). Furthermore, increased IL-1β was also found in spinal tissue of mice from the K/BxN arthritis model, and similar results were seen in cerebral spinal fluid collected from RA patients, which also had a significant reduction of IL-1 receptor antagonist (IL-1RA) and IL-4 (60).

In addition, IL-1β, IL-6 and TNF are significantly increased in the spinal cords of rats in the adjuvant-induced arthritis (AIA) model, in which microglia and MHC class II immunostaining are also enhanced (53). In the CFA monoarthritis model, the release of IL-1β and TNF in the spinal cord led to activation of the NF-κB transcriptional pathway, increasing cyclo-oxygenase (COX)-2 expression (151). COX-2 inhibition in this model suppressed chronic pain-like behaviors (151). Additionally, repressing the expression of NF- κB/p65 attenuated hyperalgesia, paw edema, and joint destruction, and reduced the overexpression of spinal TNF, IL-1β, and COX-2 (152). These findings indicate that the NF-κB/COX-2 pathway is involved in the development of the pain following peripheral tissue inflammation (151, 152).

Targeting microglia in the CAIA model by intrathecal delivery of specific inhibitors of cathepsin S (catS) and with antibodies against fractalkine (FKN), after CIA, attenuated mechanical hypersensitivity and spinal microglial response in rats (153). This suggests that catS activity and FKN cleavage are the signaling factors derived from peripheral inflammation and damage that activate microglia in the CNS (153). In addition to resident microglia, transmigration of peripheral CD11b^+^ circulating macrophages into the CNS parenchyma, due to increased vascular permeability to CNS, where they adopt microglia-like phenotype, can contribute to the development of chronic pain in inflammatory arthritis (154).

### Astrocytes

Astrocytes are the most abundant cell type in the CNS and are involved in regulating ion homeostasis, recycling neurotransmitters, preserving the BBB, and supporting neuronal function (155, 156). Astrocytes can control nociceptive output, given they are a key component of pain gating by activating Aβ-fibers (157). Suppression of astrocyte activation blocks the induction of long-term depression (LTD) in NK1R^+^ neurons at the dorsal horn and thus controls the pain inhibition exerted by Aβ-fiber stimulation (157). In addition, astroglia activation has been associated with TRPV1 activation, which contributes to increased nociceptive input from primary sensory nerves to dorsal horn neurons in inflammatory pain models (158, 159). Conversely, decreasing activation of astrocytes leads to reduced expression of this channel in the spinal tissue, which is correlated to inhibition of pain behavior in the adjuvant-induced arthritis model (160). Nevertheless, astrocytes, as microglia, also produce cytokines and chemokines, resulting in neuroinflammation that contributes to nociceptive signaling (161).

The immunomodulatory effect of astrocytes in experimental models of arthritis has different components. Astrocytes can crosstalk with T cells that infiltrate the spinal cord after CFA-induced monoarthritis and regulate the ability of infiltrating T cells to produce IFN-γ. Blockade of IFN-γ attenuates CFA-induced pain and reduces astroglia activation; however, T cell-deficient (*Rag1^−/−^*) mice can also develop mechanical allodynia and an increase in astrocyte reactivity (162). The activation of astrocytes has been detected by increased immunoreactivity of Translocator protein (TSPO) in astrocytes (163), and it has been implicated in CFA-induced monoarthritis models in rat spinal tissue (164). TSPO is a mitochondrial outer membrane protein important for steroidogenesis, cholesterol transport, immunomodulation, cell survival, and proliferation (164). In CFA-injected rats, a TSPO agonist prevents the development of mechanical allodynia and thermal hyperalgesia, providing evidence that spinal TSPO is involved in the development and maintenance of inflammatory pain behaviors in rats (164). However, TPSO is also expressed in microglia and neuronal cells, and its use as a specific astrocyte activation marker should be cautiously considered.

The reactivity of astrocytes in the spinal tissue of mice in arthritis models has been demonstrated specifically using glial fibrillary acidic protein (GFAP) staining as a biomarker. For instance, ipsilateral spinal GFAP immunofluorescence was observed and significantly increased on day 28, but not at earlier time points, in the MIA (mono-iodoacetate) OA model (63). Repeated oral dosing with nimesulide, which attenuates microglia and astrocyte activation, significantly reduced distal allodynia and GFAP immunofluorescence in MIA model of OA pain (63). Moreover, in an adjuvant-induced arthritis model, astrocytes expressing GFAP were increased in number and immunostaining intensity in the spinal cord and were associated with increased levels of cytokines such as IL-1β, IL-6, and TNF (53).

Furthermore, Huang et al. (165) described a mechanism by which IL-33 controls the expression of the above-mentioned proinflammatory cytokines (e.g., IL-6, IL-1β, and TNF), and the activation of extracellular signal-regulated kinase (ERK) and NF-κB in the spinal cord confirming findings in different pain states (166, 167). In the study, blocking IL-33 in the spinal cord significantly alleviated hyperalgesia, paw swelling, and joint destruction, suggesting a role for IL-33 in the central inflammation mediated by astrocytes in arthritis animal models (165). In addition, IL-17, which has a role in autoimmune diseases and is involved in arthritic tissues (168–170), has been found to be expressed by astrocytes and be up-regulated in CFA-induced arthritis model (171). Blocking of IL-17 significantly increased paw withdrawal thresholds and decreased NMDA receptor phosphorylation in rats injected with CFA or IL-17, suggesting astrocytic IL-17 facilitates pain (162, 171).

More evidence of astrocyte-specific mechanisms in spinal circuits in models other than the monoarthritis CFA model is needed to understand better the role of these cells in persistent pain states associated with arthritis. Furthermore, most CNS glial mechanisms studied to understand central components of arthritic pain and neuroinflammation are focused on the spinal cord even though brain and spinal glial cells are heterogeneous and can adopt region-specific phenotypes in these tissues (145, 159). In the following section, we explore the role of glial cells in the brain in the context of arthritis.

## “Arthritic brain” and pain—the role of glia cells

Around 40% of RA patients present chronic pain, which is linked to depression and anxiety as comorbidities (172, 173). Although the CNS is, to some extent, isolated from peripheral inflammatory signals by the BBB, there is evident crosstalk between the peripheral inflammatory process and the brain (173). The propagation of peripheral inflammatory signals to the brain occurs through the interaction of the peripheral immune system with the CNS myeloid cells, which include microglia and perivascular macrophages (173, 174). Among the pro-inflammatory cytokines that appear to be involved in the crosstalk between peripheral signals and the brain, IL-6 seems to be a key messenger (175, 176).

Additionally, IL-1β may play a role in brain neuroinflammation and pain perception. Increased IL-1β concentrations in cerebral spinal fluid compared to serum were found in RA patients, indicating local production of this cytokine in the CNS (60). Changes in microglial phenotype, induced by the interaction with the peripheral immune system, that can modulate neuronal activity were observed in different brain regions related to the pain experience. For instance, CIA mice showed increased density of IBA1^+^ microglia and *in situ* production of IL-1β in the hippocampus (56). Additionally, increased immunoreactivity of IBA1 in the pre-frontal cortex accompanied by an increment of brain-derived nerve factor (BDNF) expression in the hippocampus of CFA mice was reported (52). Furthermore, an increase in microglial density with a reactive and phagocytic profile (expressing CD68) was reported in the hippocampus during CIA (56). The neuroinflammation observed in the CIA mouse regulated the IGF1R signaling in the brain and neurological symptoms in RA (56). On the other hand, in a TNF mouse model of RA, a brain region-specific microglial response was observed in the cortex, striatum, and thalamus (61). The same region-specific pattern was observed in the postmortem brain of RA patients (61). Microglia modulates neuronal activity by releasing soluble factors, which include cytokines and neurotrophic factors (177). Thus, it is plausible that changes in microglia phenotype might alter the neuronal function in brain regions related to pain facilitating chronic pain development. Accordingly, it has been reported that activated microglia are involved in shaping the neuroplasticity underlying chronic pain (178) and pain-associated affective disorders (179).

This evidence supports the notion that peripheral inflammation, such as that in arthritis, is associated with immunological activation in the CNS in both humans and mice. However, as microglia, astrocytes also contribute to chronic pain development. Accordingly, it has been shown that reactive astrocytes in cortical regions associated with emotion regulation are involved in both chronic pain and chronic pain-induced emotional dysfunction (180). For instance, in the CIA model induced in mice by collagen immunization increased GFAP^+^ cells were observed in the hippocampus (56).

Additionally, astrocyte activation can cause dysregulation of glutamate and Gamma-aminobutyric acid (GABA) (181). This leads to an imbalance of excitatory and inhibitory neuronal inputs, which in turn enhances pain signals (180). Won et al. showed that cognitive impairment in the CIA animal model of RA is dependent on the astrocyte GABA-producing enzyme, monoamine oxidase-B (MAO-B) (182). The hippocampal astrocytic GABA was increased in the brains of CIA mice (182). Conversely, inhibition of MAO-B decreased hippocampal astrocytic GABA in CIA, rescued cognitive impairment, and alleviated joint swelling in CIA mice (183). This same group has shown that inhibiting MAO-B has an analgesic effect reversing mechanical allodynia present in neuropathic pain (184).

Astrocytic reactivity was observed in the medial pre-frontal cortex (mPFC), the primary somatosensory cortex (S1), anterior cingulate cortex (ACC), amygdala, thalamus, and hippocampus in several models of chronic pain (185–193). In addition, activated astrocytes in the periaqueductal grey, a well-known region involved with pain modulation, have been shown in different rodent models of chronic pain (194–197). Moreover, in different brain regions—including S1, mPFC, and ACC—astrocyte activation can lead to a dysregulation of glutamate and GABA, causing, in turn, an imbalance of excitatory and inhibitory neuronal inputs, which enhances pain signals (190, 193). Thus, since astrocytes also participate in neuromodulation (198–200), altered astrocytes may contribute to long-term neuroplasticity in nociceptive pathways in chronic pain states.

Studies in humans have demonstrated that glial reactivity is critical in chronic pain. Imaging studies using the radioligand 11C-PBR28, showed an increase in brain levels of the translocator protein (TSPO), a marker of glial activation, in people with low back pain and fibromyalgia (201, 202). The same tracer was used to detect glial activation in RA patients; however, no significant changes compared to healthy individuals were detected (203).

Although evidence suggests a link between chronic peripheral inflammation in arthritis to an induced activation of glial cells in the CNS, the role of spinal and supraspinal glial cells in the development or maintenance of chronic pain in arthritic conditions needs further elucidation. Considering chronic pain, few studies evaluate the role of astrocytes and microglia, and rather focus on the role of neurons and neuronal connections. It is noteworthy to highlight that there is bidirectional glia-neuron communication. Thus, glial cells can contribute to neuroplasticity in different pain-related brain areas, and, in turn, neurons can induce phenotypical changes in glial cells and modulate their response (204). Studies focusing on glial cells and glia-neuron interactions in the arthritic brain to either rule out or support their participation in chronic pain development and maintenance are warranted.

## Treatments targeting macrophages and glial mechanisms in peripheral and central nervous system

### Peripheral

**Figure 2:**
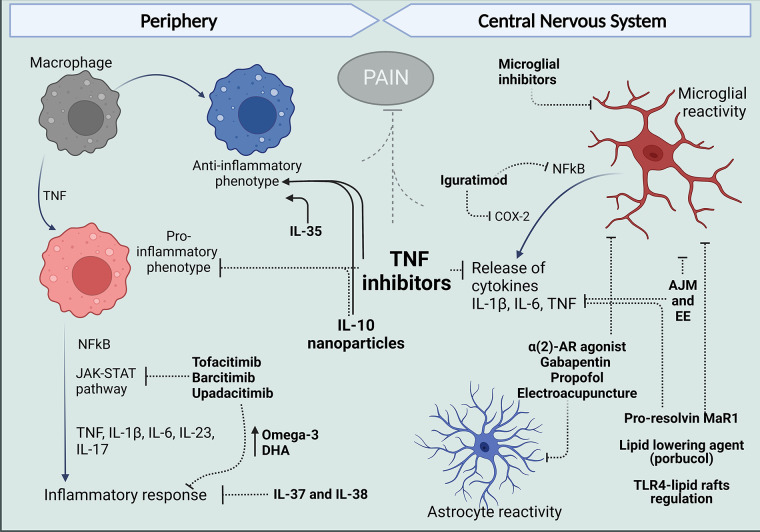
Treatments that target peripheral and central mechanisms involving macrophages and glial cells for the control of neuroinflammation and pain in RA. In The periphery, targeting TNF with antibodies such as ADA and anti-inflammatory cytokine administration like IL-10 have reduced the release of pro-inflammatory cytokines and reduce polarization of macrophages in the synovium to an M1 phenotype and, in contrast favor polarization to the anti-inflammatory M2 profile. Different inhibitors of JAK kinases and treatments with IL-37 and IL-38 have been reported to increase anti-inflammatory Omega-3 and PUFAs and reduce cytokine release and inflammatory response. Centrally, evidence in arthritic models suggests articular joint movement and environment can affect glial reactivity as well as alpha 2-AR agonist, gabapentin, propofol, and electroacupuncture. Pro-resolving lipids and lipid-lowering agents have been shown to inhibit glial increased reactivity inflammatory response and produce analgesia. And in models different from arthritis, DMARDs have been shown to have an effect in microglia, targeting inflammatory mechanisms described for these cells in the spinal cords and brains of mice models of arthritis. Figure created with Biorender.com. ADA, adalimumab; AJM, articular joint movement; EE, environment enrichment; Mar1, maresin-1; DHA, docosahexaenoic acid.

As described in previous sections, macrophages and glia produce effector molecules that participate in the pathophysiology of RA, and several of those pathways and proteins are now targeted for therapeutic purposes (205). These interventions are capable of modulating macrophage and glial cell activity involved in ongoing pain perception and have a significant impact on disease outcome, given the role of these cells in the disease pathology (Figure 2).

Pharmacological therapies such as NSAIDs, glucocorticoids, and disease-modifying anti-rheumatic drugs (DMARDs) have been shown to regulate macrophage activity. Low doses of the NSAID Naproxen, for example, when added to macrophages, both pre-and post-activation with LPS or hyaluronan fragments, significantly reduced NF-kB activity, and prostaglandin E_2_ (PGE2) release (206). In contrast, acetaminophen (APAP), another over-the-counter pain relief drug, can increase the release of pro-inflammatory cytokines IL-1β and TNF by macrophages treated in-vitro *via* CYP1 cytochrome and p38, ERK and JNK MAPK pathways (207). On the other hand, the sustained release of Ibuprofen through nanoparticles locally decreased the edema and the concentration of IL-6 and TNF in CFA-induced arthritis (208). Although NSAIDs are able to improve active RA symptoms, DMARD agents have been shown to alter the disease course (209, 210), providing overall physical health benefits and increased pain control.

Kinases mediate the induction of cytokine production in different inflammatory responses reported in RA (211). In addition to MAPKs, the Janus kinase (JAK)-STAT pathway is a common signaling pathway that regulates biological processes induced by macrophages, including cytokine release (212–214). Yet, inhibition of the JAK-STAT pathway by inhibitors, such as Ruxolitinib and Tofacitinib, increases TNF, IL-6, and IL-12 secretion in mouse bone marrow-derived macrophages (BMDM) stimulated with LPS (215). On the other hand, a translational study showed that JAK1-mediated interferon and IL-6 release play key roles in the synovial response and described how targeting this mechanism with Tofacitinib reduced MMPs and interferon-regulated gene expression in the synovium resulting in clinical improvement (212). Furthermore, treatment with JAK inhibitors demonstrated improvement in pain (inflammatory and of other types) and physical function in RA studies (216). The pain reduction effect of JAK inhibitors has been linked to increased levels of omega-3 fatty acids and DHA in patients treated with JAK inhibitors (217), and studies revealed that omega-3 PUFAs and DHA might be effective in reducing pain in RA patients (218).

TNF is produced locally in RA joints by synovial macrophages and is a critical cytokine that mediates joint damage and destruction (219–221). Biologic agents that target TNF are widely used in the treatment of RA (205, 222) and different constructs have been engineered (205, 222). TNF inhibitors include: (i) Etanercept (ETA), a soluble TNF receptor-Fc immunoglobulin fusion construct; (ii) infliximab, adalimumab, and golimumab, monoclonal antibodies; and (iii) certolizumab pegol, an anti-TNF binding domain with a polyethylene glycol moeity (222). Although etanercept and adalimumab (ADA) both block soluble TNF, the monoclonal anti-TNF agents have broader clinical efficacy. Both ETA and ADA act through similar mechanisms: ETA and ADA bind to soluble and receptor-bound TNF molecules. However, ADA blocks soluble TNF and membrane bound TNF trimers simultaneously, which allows the blockade of multimeric complexes while ETA seems to bind only to a soluble TNF (222–224). Thus, the structural differences between ADA and ETA may implicate different outcomes (75). Interestingly, evidence shows that RA patients treated with ETA for 6 and 12 weeks had an up-regulation of IFN-γ and TNF production from CD4^+^ and CD8 (225). Given the constantly renewed inflammatory pathology in RA, the suppression of TNF signaling promoted by ADA might induce more effective management for certain disease manifestations, reflecting better clinical outcomes in comparison with ETA.

RA patients had enhanced *TNF* mRNA expression in monocytes and macrophages, defects in differentiation into M2-phenotype induced by M-CSF, and a propensity for preferential maturation toward M1-like macrophages that may contribute to synovial inflammation (219). Moreover, this defect correlated with *TNF* mRNA transcript levels and microRNA 155 (miR-155) expression and was partially reversed by ADA (221). In this same study, monocytes collected from healthy donors transfected with mir-155 showed a decrease in M2-like markers, and transfection of RA monocytes with antagomir-155 allowed restoration of M2-like polarization (221).

Furthermore, a longitudinal study (221) showed that ADA, but not ETA, restored the impaired M2-like polarization in circulating monocytes induced in RA. Additionally, RNA-sequencing analysis revealed that RA patients have ERK/MAPK, PI3K/AKT, STAT3, and GM-CSF signaling significantly up-regulated as well as pro-inflammatory cytokines or transcription factors involved in macrophage polarization, such as TNF-IFN-γ/α/β -IL-1β-IRF5-TP53-STAT1-ERK-p38MAPK-NFkB, and a decrease of IL-10 and SOCS-1 (221). On the contrary, in patients receiving ADA, this macrophage pro-inflammatory response was decreased, and the transcriptomic signature of M2-related transcripts such as SOCS-1, IL-10, CEBPβ, and c-MYC was restored (221). Despite these promising data, TNF inhibitors are more effective in patients with short-term disease and have less effect on pain in patients with chronic diseases (226–228).

Tocilizumab is a humanized monoclonal antibody that acts as an IL-6 receptor antagonist and is used as monotherapy or in combination with conventional synthetic DMARDs (csDMARDs) in adults with moderate to severe RA (229). Tocilizumab has been used for treatment in patients with inadequate responses to TNF inhibitors and has resulted in rapid and sustained improvements in pain and other patient-reported outcomes (228, 230).

A therapeutic alternative to blocking inflammatory cascades in RA might be the delivery of interleukins which have shown promising results in regulating macrophage function (231–237). Alginate nanoparticles carrying plasmid DNA encoding IL-10 were successful in reprogramming macrophage phenotype balance of synovial macrophages in CFA—arthritic rats. The treatment with IL-10 also reduced systemic and joint tissue pro-inflammatory cytokines (TNF, IL-1 β, and IL-6) expression and prevented the progression of inflammation and joint damage (231). The potential for IL-34 and IL-35 has also been described. While IL-34 favors monocyte survival, proliferation, and differentiation of macrophages, IL-35 promotes TNF-induced apoptosis of FLS and an anti-inflammatory M2-like macrophage, inhibiting inflammation in the murine model of CIA (232–234). Additionally, there is substantial evidence for the roles of IL-37 and IL-38 in counteracting immune responses in RA (235, 236). IL-37 alleviated joint inflammation in RA patients (170). Complementary, CIA mice developed robust levels of IL-17 and marked joint inflammation, which was dramatically reduced by systemic delivery of IL-37, indicating a pivotal role of IL-37 in reducing IL-17 mediated joint inflammation (170). In the work of Boutet et al. (237), clinical inflammatory scores were significantly decreased after IL-38 injection in joints of mice with CIA, accompanied by reduced macrophage infiltration and lower expression of IL-17, IL-23, and TNF. Conditioned media from an M1 macrophage line that overexpressed IL-38 reduced the inflammatory profile in FLS and macrophages from RA patients. Despite these anti-inflammatory effects, IL-38 overexpression in the CIA model had no effect on cartilage or bone destruction (237) (Figure 2).

### Central

Proinflammatory cytokines released by glial cells in the CNS that lead to activation of major pain pathways, from the dorsal horn and supra-spinal pathways to the somatosensory and other higher cortical centers, are targets for intervention with anti-inflammatory agents. For instance, brain-penetrating corticosteroids (e.g., prednisolone, triamcinolone, and others) have been hypothesized to exert an analgesic effect by downregulating neuroimmune processes underlying chronic pain (238, 239). This central effect is supported by mounting *in vitro* data indicating that glucocorticoid treatment reduces the production of inflammatory cytokines by microglial cells (240, 241). Regulatory effects of steroids are also observed in acute and chronic exposure of astrocytes to cortisol. Chronic cortisol exposure in mice regulates mRNAs in multiple brain regions that are also regulated by glucocorticoids in astrocytes *in vitro* and by acute cortisol exposure *in vivo*, including mRNAs associated with known astrocyte function, such as glutamate reuptake and metabolism and gap junction connectivity (242, 243). However, deletion of mineralocorticoid receptors in myeloid cells *in vivo* has been shown to attenuate reactive microglia in the CNS and instead induce an anti-inflammatory phenotype, and ameliorated neuroinflammation. This suggests a detrimental effect of the use of corticoids (244), which can contribute to and explain the adverse effects of corticosteroids. Thus, they are not regarded as an appropriate long-term option for the treatment of chronic arthritic pain (239).

Few DMARDS have been described to control microglia inflammatory responses but in other disease contexts. For instance, Iguratimod which was originally reported to be a selective inhibitor of COX-2 that inhibits the synthesis of pro-inflammatory prostaglandins (PGs) (245, 246) also acts as an immunomodulatory agent and suppresses the production of pro-inflammatory cytokines, such as interleukin IL-1β, IL-6, IL-8, and TNF, by activated monocytes/macrophages *in vitro* (247). In a model of Experimental autoimmune encephalomyelitis (EAE), Iguratimod reduced infiltration of immune cells into the spinal cord and suppressed macrophage and microglia activation in the parenchyma at the acute and chronic stages of EAE. In this study, therapeutic administration of Iguratimod after the onset of clinical symptoms ameliorated the clinical severity of chronic EAE and reduced microglial activation, NF-κB p65, and COX-2 expression in the spinal cord (248).

Additionally, the TNF inhibitor adalimumab (ADA) significantly reduced microglial activation and reversed pro-inflammatory IL-1β, IL-6, IL-12, INF and TNF cytokine levels in a model of vascular dementia in rats (249). Additionally, adalimumab treatment suppressed NF-κB, activity and ameliorated memory impairments (249). This suggests that the effects of biologics, deemed to be peripheral due to poor BBB penetration, could also modify central glial inflammatory responses and thus contribute to the control of central aspects of arthritic pain development.

Preclinical data also show that controlling microglia itself can alleviate arthritic pain. For instance, intrathecal delivery of microglial inhibitors, such as a catS inhibitor, or an FKN neutralizing antibody, attenuated mechanical hypersensitivity and spinal microglial response in rats with CIA (153). Furthermore, different strategies have been proven to reduce arthritis pain in rats subjected to the CFA-monoarthritis model, by targeting microglia and astrocytes reactivity (250–253). These preclinical studies show evidence that blocking spinal glial activation is involved in the analgesic action of dexmedetomidine [a highly selective α(2) -adrenoceptor (α(2) -AR) agonist], gabapentin, propofol, and electroacupuncture. In these studies, reduced spinal astrocyte and microglial marker reactivity were accompanied by suppressed proinflammatory cytokines, IL-1β, IL-6, and TNF in spinal tissue (251–253). Propofol inhibited CFA-induced microglia activation and neuroinflammation (TNF, IL-6, and IL-1β expression) *via* activation of ERK1/2/NF-κB signaling pathways (250).

Additionally, analgesic effects with articular joint movement (AJM) treatment have been reported in a model of persistent inflammation. AJM did not altered cytokine levels at the inflammatory site, but centrally, AJM reduced the levels of pro-inflammatory cytokines IL-1β and TNF in the spinal cord, which suggests a central neuro-immunomodulatory effect (254). This data corroborates other pre-clinical studies showing that non-pharmacological interventions such as environmental enrichment (EE) can modulate the nociceptive and inflammatory responses in arthritis models. For instance, in the mouse model of arthritis induced by CFA, EE diminished the IBA1 immunopositivity in the pre-frontal cortex, and reduced, to some degree, GFAP^+^ reactive astrocytes in hippocampus (52).

Moreover, lipid mediators exert different effects on macrophages, and pro-resolving lipid mediators have been described to have an antinociceptive effect mediated by reduced inflammation (255, 256). For example, in addition to the peripheral effect induced by pro–resolving Maresin-1 (MaR1) to control macrophage numbers in the DRG in the K/BxN serum transfer model, MaR1 reduced microglial cell activation, NF-κB activation, IL-1β, and TNF production in the spinal cord, all of which were correlated to the analgesic effect (257). Additionally, lipid-lowering agents also exert analgesic effects in CFA-monoarthritis in mice. Probucol, a synthetic polyphenolic compound, inhibited CFA-induced hyperalgesia by attenuating NF-κB pathway and reducing microglia and astrocyte activation in the spinal cord (258). In addition, disruption of spinal microglial lipid rafts and TLR4 signaling by cholesterol efflux mechanism induced by apoA1 binding protein (AIBP), reduced the pro-inflammatory gene profile in microglia and cytokine levels in the spinal tissue alleviating neuropathic pain (259). This mechanism may well be targeting spinal microglia in arthritis (Figure 2).

## Future directions and perspectives

This review highlights the importance of the macrophage phenotype in the peripheral nervous system and the emerging roles of these phenotypes in the synovium and DRG. The review of glial cells in the CNS additionally shows there is a clear need to expand the pre-clinical and clinical studies aiming to understand and target spinal glial cells and central pathways given the data suggesting their critical contribution to neuroinflammation and pain in arthritis. We want to emphasize several issues for future direction and in conclusion.

Although not highlighted in the text, aging is a critical factor for arthritis onset. Several works support the premise that age-related senescence of the innate immune system is a major mechanistic pillar responsible for the malfunctioning of cell damage sensors and their induced response feedback (260–262). Immunosenescence creates a vulnerability scenario where chronic low-grade inflammation (characterized by increased serum levels of inflammatory cytokines such as TNF, and IL-6) leads to a systematic tissue injury and joint degeneration that may accelerate RA development and morbidity (260, 263). Moreover, it has been described that microglia can adopt a pro-inflammatory phenotype with age that aggravates neurodegeneration (264). However, if the phenotype of microglia or other glial cells and macrophages changes with aging during the curse of arthritis is not clear and deserves its own review.

The evidence gathered here represented, in large part studies from male subjects. However, substantial evidence shows that women and men diverge on pain regulatory mechanisms linked with several pathological conditions (265) including arthritis (266). In this sense, multiple studies indicate that a sexually dimorphic immune response might contribute to the pain phenotype in many pre-clinical models (267). These differences have been attributed to different cell types and cytokine mediators (268). For example (268), showed that IL-23-induced pain behavior in female mice is promoted by estrogen but suppressed by androgen, suggesting the involvement of sex hormones in IL-23-induced pain. However, IL-23 acts indirectly and requires IL-17A release from macrophages to evoke mechanical pain in females (268). Estrogen receptor mediation of the pain phenotype evoked by IL-23 and IL-17 may be a hint for the different pathological outcomes observed in women and men regarding arthritis. In addition, despite data suggesting differential macrophage expansion between sexes in different pain models (112), and the well-described role of estrogen in macrophage polarization (269, 270), the role of these sex differences in macrophage imbalance in arthritis remains unknown. Studies to fill this gap are warranted and will be beneficial in leading to effective therapies for arthritis pain for all patients.

While the bulk of this review has focused on the non-neuronal cell populations, the observation that innate immune receptors, such as TLR4, are present on DRG nociceptive neurons implies that immune mechanisms and activation by TLR4 agonists could directly enhance neuronal excitability (271–273). Future work characterizing the nature of the interaction between TLR4, and other receptors within the lipid rafts on spinal microglia, DRG macrophages, and DRG nociceptors will likely prove interesting in defining the contribution of their interaction in generating the arthritic pain phenotype.

Mechanisms reviewed here described the role of macrophages and glial cells in the peripheral terminal and central processing. However, we want to highlight the relevance of macrophages and glial effects on the DRG, given the changes in the input/output function of the afferent traffic generated by injury and inflammation at this site. Notably, the DRG is outside of the blood-brain barrier, and circulating factors, such as immune complexes, can directly influence the excitability of this system (106–108). Conversely, this access provides a preferred route for therapeutic interventions.

The perception of pain reflects upon the role played by higher order systems, e.g., pain is in the brain (274, 275). However, it is equally clear that the pain response mediated at higher centers is driven by the input that it receives from the afferent limb of the somatosensory system (274, 276, 277). This review emphasizes the robust mechanisms that can regulate the input generated by inflamed or injured peripheral structures. Regulating the content of the message received by the brain serves to diminish the response organized at the supraspinal level (e.g., the pain state).

In conclusion, pain control in RA, as its pathophysiology, is complex and multifactorial. Strategies that focus on the phenotype of innate immune cells in the periphery and along the neuraxis may prove relevant. A need for a deeper understanding of spinal and supraspinal glial mechanisms will also contribute to the discovery and validation of new targets for pain treatment in this disease.
